# Medical News and Miscellany

**Published:** 1892-07

**Authors:** 


### Medical News and Miscellany.

Dr. S. Leard Keoun has removed from Comanche to Waco.

Dr. T. D. Wooten is spending the summer in Arkansas.

Dr. A. B. Holder is not merely a beholder, or speculator, but an active operator at the Memphis Clinics.

They say Pasteaur is sick with an attack of cholera. He has been down on it a long time; now he is down with it.

Dr. S. E. Hudson, of Round Rock, Texas, has purchased an interest in the *Texas Sanitarian* and will remove to Austin.

The notorious "electric" doctor, Yowell, a long time at

Greenville, Texas, was killed in Denison, Texas, June 27th, by Editor Harris, of Greenville, whose wife Yowell had traduced.

---

The JOURNAL is pained to note the serious illness of Mrs. Bennett, wife of Dr. T. J. Bennett, Managing Editor of the *Texas Sanitarian.*

---

Wanted.—Somebody to stamp out from medical literature the phrase "stamp out," as applied to suppressing an outbreak of disease. It has become as stereotyped as "busy practitioner."

---

Dr. W. A. Adams, of Fort Worth, Surgeon of —— Regiment, Texas Volunteer Guard, was detained at home by sickness, and prevented from being with his regiment during the encampment.

---

Dr. D. R. Wallace, of Waco, has gone to San Francisco, whence he will make an intended tour of the Pacific slope, going up as far as Alaska. Here he will spend some time, and will return to Waco by October 1st.

---

Dr. H. H. Harrington, Professor of Chemistry in the Agricultural and Mechanical College at Bryan, Texas, is to be married August 10th prox., to Miss Florine Ross, daughter of ex-Governor Ross, President of the College.

---

St. Louis College of Physicians and Surgeons have their announcement in this issue. Please read it. Of especial interest to physicians who have sons or brothers studying medicine. Dr. G. W. Cale, 2600 Gamble street, is the Secretary. Address him for particulars.

---

Manufacturers and advertisers generally will find this, the exponent of rational medicine—the favorite home journal with the Texas doctors—the very best medium for bringing their products to the attention of a wide circle of intelligent readers. Send for rates.

---

Robert A. Barnes, of St. louis, recently deceased, bequeathed about $900,000 for the erection and maintenance of a hospital in that city. It will be conducted under the auspices of the

Southern Methodist church, but will dispense its charity regardless of creed or nationality.

---

"A Live Journal."—Battle & Co., in renewing their advertisement for the *eighth consecutive year*, cheerfully stand a rise of 30 per cent. on past rates, and write, "Yours is a live journal, and all manufacturers should advertise in it."

That tells the tale. See their new ad, and see the other twenty new ones in this issue.

---

Quarantined Against Yellow Fever.—The steamer Enchantress from Santos and Pernambuco was detained at quarantine July 17. During the voyage Captain Hammond and Purser Foster were stricken with yellow fever, died and were buried at sea. Immediately following these deaths Steward Wolmsley and Second Engineer Pottinger died on the voyage.

---

An advertisement in the JOURNAL of a practice wanted elicited twenty-three replies; an advertisement of a practice to sell had thirteen inquiries. Sales affected in both cases. Here is your opportunity, doctor, either to buy or sell. Such ads. are charged $1.50 each for two or three insertions, or $2.50 for one insertion, money with order. Address Editor DANIEL'S TEXAS MEDICAL JOURNAL, Austin, Texas.

---

Doctor, show this copy of the JOURNAL to your friends,—tell them of its struggles and triumphs, and endeavor to secure their support. We earnestly invite your influence in its behalf, as well as your cordial support and co-operation. The possibilities of the JOURNAL are practically unlimited; it should be made, and can be, with your aid, the leading journal in the South,—and as Texas leads in all else, that is our ambition.

---

We take pleasure in offering the *amende honorable* for an unjust reflection on the standing of Dr. Emory Lanphear, of Kansas City, Mo., which appeared in our last issue. We were incautiously led into an error which we much regret by an item found in the columns of an unhealthy exchange from the Southwest [Dallas.—ED.] Our quotation was unfortunate and ill-advised, and we hope Dr. Lanphear will pardon our indiscretion which, we are sure, did him much injustice.—*Atlanta Medical Journal.*

**A Texas Favorite.**—By reference to the annual announcement of the University of Louisville Medical College for the fall and winter session of 1892–3, we note that their class last year numbered four hundred and eight (408) matriculants, of whom fifty-two went from Texas. The Texas students love this school, and many of them carry off the prizes. Many of the alumni are leading Texas practitioners, doing remunerative practice, respected and honored by all who know them. We are pleased to note the continued prosperity and advancement of this popular old institution. For catalogue address the Dean, Dr. J. M. Bodine. He was a long time a Texas practitioner.

---

**An Opening.**—Doctor, if you are seeking a good location to practice and conduct a drug business, you can hear the particulars of a rare chance by addressing me. Income over $2000 a year; no bad debts; everybody pays up, as it is in the midst of a good cattle and farming section, with educational, social and religious advantages. Only $1200 required. Reason: Owner wants to go to a city. A prosperous town in northwest Texas. Will sell drug business, office fixtures, etc., and can turn over practice and influence. Only one other physician in the county.

Address: Dr. C. M. S., care DANIEL'S TEXAS MEDICAL JOURNAL, Austin.

---

**Big Bills for Witnesses.**—Dr. G. De F. Smith has filed a claim against the city for $500 for services as an expert witness for the people in the trial of Carlyle W. Harris, the medical student, for the murder of his wife, Helen Wilson Potts Harris.

Professor Witthaus, the chemical expert who made the analysis of the contents of the dead woman's stomach, has filed with the district attorney a bill of $5,000 for that service.

Dr. Allan McLane Hamilton, another expert witness in the case, has collected a bill of $1,500 for his services, and other bills from expert witnesses have been filed, which bring the total cost of the expert testimony for the people up to $9,000.

The bills of the medical experts who testified in the trial of E. M. Field aggregate $4,000, and none of them has yet been paid. —*New York Evening Snn.*

---

**Salol for Gonorrhœa.**—Dr. E. C. Underwood says that salol can reduce the duration of gonorrhœa to the lowest limits. This method consists in the regular employment of from forty to sixty grains of salol through the day. I order my patients to have four doses of from ten to fifteen grains each, taken immediately on rising in the morning, at 11 o'clock a. m., 4 o'clock p. m., and the last thing on retiring to bed at night. This is ordered in a powder or compressed tablets. Having known that many of these tablets passed through the intestinal canal without being absorbed, and in the form they were administered, I am now using the drug in the powder form. It is tasteless and is not complained of by patients. The dose is begun, unless this patient shows that the drug disagrees with him, with sixty grains a day, and continued until the discharge has become very meager. Then it is gradually lessened. The author claims that better results follow this method than any other.—*West. Medical Reporter.*

---

**Intolerance.**—The egotistic and notorious Fisher, of San Antonio, formerly of Austin, editor of the *Southern Journal of Homeopathy*, in a loud wail, in his June number, denounces the regular medical profession of San Antonio as "bigoted, ignorant and intolerant." He thinks he is the only "white man" in San Antonio, and the embodiment of intelligence. *We* think he is a most intolerant and intolerable bigot, too conceited and small for discussion in a journal; but the San Antonio *Light* takes him to task for his slanderous utterances, and says that in denouncing the regular physicians of San Antonio as ignorant, he reflects upon the intelligence of the citizens in employing them; and refutes his charges by citing the presence, among those "bigoted and ignorant and intolerant" doctors, of such men as Drs. Cupples, Herff, and others,—men of world-wide reputation as surgeons and physicians. The only comment the JOURNAL has to make on Fisher's *whine* is, that the presence in San Antonio of Fisher himself, and eleven others of his sort,—homeopaths, eclectics, electric—healers, etc.,—disproves his charge of intolerance. Any community that can "tolerate" Fisher, cannot be called "intolerant."

---

**Barnes Medical College.**—Mr. Robt. A. Barnes, who recently died in the city of St. Louis, Mo., left in his will a provision for a hospital to bear his name, and to be under the charge and supervision of the Methodist church. He endowed this institution upon a scale superior to that of any similar hospital in the country, and his gift, amounting to more than a million dollars, was greater in amount than has ever before been contributed by a private citizen. During the sixty-two years that he resided in the city of St. Louis, he was a prominent figure in many of its most important corporations, and contributed largely of his means to many charitable and educational institutions. He is credited with building no less than fifteen churches in the southern part of this State. As an expression of appreciation, and as an honor to the memory of Mr. Barnes for his noble charities, the Barnes Medical College is named. The institution is under the control and management of a Board of Trustees composed of upright, influential business men. Its Faculty is composed of men of faultless integrity and of experience as instructors, and who are in line with the best element of the medical profession, and who are in hearty accord with the trend of public and professional sentiment with reference to advanced medical education. Among the teachers are Drs. Chas. H. Hughes, A. M. Carpenter, James T. Jelks, Wm. Dickinson, John W. Vaughn, Pinckney French, Frank D. Wright, S. C. Martin, and others.

---

**Roster of Medical Officers.**—The following is the roster of the medical officers of the division of the Texas Volunteer Guard State militia now in camp at Austin:

R. M. Swearingen, Austin, Surgeon-General.

F. E. Daniel, Austin, Acting Surgeon-General.

F. C. Ford, Nacogdoches, Surgeon First Regiment and acting Medical Director.

J. H. Reuss, Cuero, Acting Surgeon First Regiment.

C. M. Ramsdell, Lampasas, Acting Surgeon Second Regiment.

W. E. Brown, Coleman, Acting Surgeon Third Regiment.

W. A. Duringer, Fort Worth, Surgeon Fourth Regiment.

W. L. Marshall, Longview, Surgeon Fifth Regiment.

W. B. Gearhart, Henrietta, Surgeon Sixth Regiment.

G. W. Larendon, Houston, Surgeon Brigade Cavalry.

W. A. Lockett, Brenham, Surgeon Battalion Artillery.

In addition to the above several companies have surgeons; but they have no official position. Amongst those present at camp are:

Dr. Matt Smith, Austin, Surgeon of the Governor's Guards.

Dr. C. L. Swearingen, Center, Texas, Surgeon Shelby Rifles.

Dr. Garrett, Sulphur Springs, Surgeon to Sulphur Springs Co.

Dr. J. D. Westervelt, Jr., Corpus Christi Light Guard—detailed as Hospital Sergeant.

Dr. F. R. Ramsdell, Lampasas, Surgeon to ——— Company, and acting as Senior Hospital Sergeant.

Dr. Haynes, Mobile, Texas, detailed as Hospital Sergeant.

The medical staff is not yet fully organized. We learn that Dr. Grizzard, Dr. Duffau and Dr. Dart, Regimental Surgeons, have resigned.

### SOUTHEAST TEXAS MEDICOS.

ORANGE, TEX., July 15.—The Southeast Texas Medical Society held its regular quarterly meeting in the Knights of Pythias hall, Beaumont, Texas, Tuesday, July 12. Dr. A. N. Perkins presided, Dr. F. Hadra, Secretary. There were present Dr. A. N. Perkins of Sabine Pass, Drs. B. F. Calhoun, C. Y. Thomson,

Price and Blewitt of Beamont, Drs. Sholars and F. Hadra of Orange, Dr. Cruse of Kountze, Dr. T. B. Selman of Village Mills and Dr. Yates of Salem.

The minutes of the previous meeting were read and approved.

Dr. Calhoun read an interesting paper on typhoid fever, which led to an animated discussion participated in by Drs. Perkins, Sholars, Price, Cruse and Hadra.

Dr. Price then read a paper on la grippe which evidenced much labor, and knowledge of the subject. Owing to the lateness of the hour discussion thereon was postponed until the next meeting at which Dr. Roberts will also read his paper on "Hæmaturia Miasmatica."

After the transaction of routine business the Society adjourned to meet again at Beaumont on the first Tuesday of September.

---

The Special Attention of the readers of this issue of the JOURNAL is called to the fact that Eugene Von Boeckmann, at 913 Congress Avenue, is prepared to bind the last volume of the JOURNAL in the best style, and at the lowest price for good work. Music, magazines and periodicals bound, and books re-bound. All in the best style. Give him a call, or drop him a postal for prices, etc.

